# Use of antipsychotic drugs during radiotherapy in adult cancer patients in Korea: a nationwide retrospective cohort study based on the national health insurance service database

**DOI:** 10.1186/s13014-024-02558-8

**Published:** 2024-11-29

**Authors:** In Gyu Hwang, Song E Park, Sun Mi Kim, Dae Ryong Kang, Tae-Hwa Go, Se Hwa Hong, Yong-Chan Ha, Shin Young Park, Hyunho Lee, Jin Hwa Choi

**Affiliations:** 1 Department of Internal Medicine, Chung-Ang University College of Medicine, Seoul, Korea; 2 Department of Psychiatry, Chung-Ang University College of Medicine, Seoul, Korea; 3 Department of Medical Informatics and Biostatistics, Yonsei University Wonju College of Medicine, Wonju, Republic of Korea; 4 Department of Orthopaedic Surgery, Seoul Bumin Hospital, Seoul, South Korea; 5 Anticancer Strategy Research Institute, VSPharmTech Co., Ltd., Seoul, South Korea; 6 Department of Radiation Oncology, Chung-Ang University College of Medicine, 84 Heukseok-ro, Dongjak-gu, Seoul, 06973 Republic of Korea; 7 Department of Precision Medicine, Yonsei University Wonju College of Medicine, Wonju, Republic of Korea

**Keywords:** Cancer, Radiotherapy, Antipsychotic drug

## Abstract

**Background:**

Antipsychotic drugs (APDs) are used for treating mental illnesses and are also used by cancer patients. This study aimed to evaluate APD use in adult cancer patients who received radiotherapy (RT) in South Korea and assess the effects of APD use during RT on survival.

**Methods:**

This retrospective cohort study utilized the National Health Insurance Service database database of Korea. We included adult cancer patients who underwent RT or chemotherapy (CTx, cisplatin, or 5-Fluorouracil) between 2010 and 2020. The APDs included in the analysis were aripiprazole, quetiapine, olanzapine, risperidone, haloperidol, and chlorpromazine.

**Results:**

Overall, 725,897 patients received RT, and 115,500 received concomitant chemo-radiotherapy (CCRT). Of them, 41,118 (5.6%) took APDs during RT, and 8,129 (7%) took APDs during CCRT. Overall, 27,789 (67.58%) patients who took APDs during RT were men, and 28,004 (68.2%) were aged ≥ 60 years. The most frequently used APD during RT was quetiapine (64.93%). Patients who took APDs during RT and during CCRT had higher mortality rates (HR: 3.45 and 1.72, *p* < 0.0001, respectively) compared to the non-APD patients. Of the patients who used APDs during RT, patients accompanying psychiatric diagnosis, taking high-dose APD, and taking APD for more than 3 months had lower mortality than patients without psychiatric diagnosis, taking low-dose APD, and taking APD for less than 3 months, respectively (HR: 0.88, 0.87 and 0.80, respectively, *p* < 0.0001).

**Conclusions:**

Only 5.6% of patients who underwent RT used APDs, and quetiapine was the most frequently prescribed APD during RT. The use of APD during RT may adversely affect survival. Further studies are required to elucidate the effects of APDs on cancer patients.

**Trial registration:**

This study is retrospectively registered.

**Supplementary Information:**

The online version contains supplementary material available at 10.1186/s13014-024-02558-8.

## Background

Cancer remains a major cause of global mortality, accounting for approximately 19 million newly diagnosed cases and 10 million cancer-related fatalities in 2020. Projections indicate a further rise in cancer incidence in the coming years, with an estimated global incidence of 28 million new cancer cases for the year 2040 [1]. Cancer and its treatment are known to impact patients’ mental health significantly. Reports suggest that a minimum of 30% of cancer patients fulfill psychiatric diagnostic criteria, with approximately 10% experiencing anxiety disorders, around 30% experiencing depression or adaptive disorders, and 25–59% experiencing sleep disorders [2, 3]. Antipsychotic drugs (APDs) are useful in treating psychiatric disorders, including bipolar disorder, psychotic disorder, depressive disorder, and delirium. Active treatment utilizing APDs is also employed to address the psychiatric disorders in cancer patients [2, 4]. APD use in cancer patients varies based on several factors, including the type of cancer, treatment protocols, and the presence of psychiatric symptoms. APDs are not typically prescribed as a standard treatment for cancer; however, they may be used to manage certain symptoms arising from cancer or its treatment, such as delirium, agitation, or psychosis.

Repurposing drugs for various diseases have proven to be a cost-effective and time-saving alternative to the traditional process of developing new drugs. APDs are known for their anti-cancer properties, and recently, there has been growing interest in their potential role in cancer treatment. Recent studies have shown a decreased incidence of certain types of cancer in patients with schizophrenia who are treated with APDs [5, 6], indicating a potential anticancer effect. APDs also been found to have the radio-sensitizing effect in experimental studies [7, 8].

Radiotherapy (RT) plays a pivotal role in the treatment of cancer and is administered for curative and palliative purposes. Typically, RT is delivered in multiple sessions or fractions spread over several weeks. The duration of RT can vary depending on several factors, including the type and stage of cancer, the treatment goals, and the specific treatment plan determined by the radiation oncologist. Since RT is delivered over a long period, patients prescribed APDs will have to take it while receiving RT.

No studies have analyzed the effect of APDs on treatment outcomes during RT. Additionally, there are no reports on the use of APDs by cancer patients in Korea. This study aimed to evaluate the use of APDs by adult cancer patients in South Korea who were receiving RT. In addition, we assessed the effect of APD use during RT on survival.

## Methods

### Data sources and selection

In this retrospective cohort study, we utilized data obtained from the National Health Insurance Service (NHIS) database, which encompasses medical expense claim data for the entire South Korean population. We obtained the claims data of patients treated with RT or chemotherapy (CTx) with diagnostic codes C00 − C97 based on the 10th edition of the International Classification of Diseases (ICD-10) from NHIS between 2010 and 2020. The CTx drugs were limited to 5-fluorouracil (5-FU) and cisplatin (CDDP), which are mainly used for concomitant chemo-radiotherapy (CCRT). The claims data included prescriptions for APDs, including aripiprazole, quetiapine, olanzapine, risperidone, haloperidol, and chlorpromazine, as well as prescriptions for RT, 5-FU, and CDDP. The claims data included age, gender, Charlson Comorbidity Index (CCI), and diagnostic codes for chronic disease and psychiatric disorders. The diagnostic codes included in the analysis besides cancer were hypertension (I10-I15), diabetes (E10-E14), dyslipidemia (E78), chronic renal disease (N17-N19), stroke (I60-I69), peripheral vascular disease (I70-I79), myocardial infarction (I21, I22), anemia (D50-53, D55-59), bipolar disorder (F30, F31), psychotic disorder (F20-F29), depressive disorder (F32-F34), delirium (F05), and other psychiatric disorders (F00-F99). Medical history included all patients with diagnostic history for the entire period and psychiatric history included patients with diagnostic history before or during RT. This study utilized research data prepared by NHIS and was analyzed by experienced statistical experts. This study was performed after approval from the Institutional Review Boards of Chung-Ang University Hospital (CAUH IRB No.2202-022-19407).

### Operational definition

In this study, data recorded between 2010 and 2020 were provided by NHIS. Patients with RT-related procedure codes were included in the RT group, while those with 5-FU/CDDP plus RT claims were included in the CCRT group. In all patients with RT claims, APDs were considered prescribed and taken if they were claimed at least once. If the RT period overlapped with the claimed APD period, it was considered that APD was taken during RT. In all patients with CCRT claims, APD was considered prescribed and taken if APD was claimed at least once. If the CCRT period overlapped with the claimed APD period, it was considered that APD was taken during CCRT. Patients who underwent two or more RT sessions with interruptions and the same diagnostic code were counted as one patient. Patients who took APDs were categorized as “APD patients,” while those who did not take APDs were categorized as “non-APD patients.”

The APD patients were divided into high-dose and low-dose groups based on the prescribed dose. The APD doses were expressed as equivalent to chlorpromazine 100 mg/day and included quetipine 60 mg/day, olanzapine 3 mg/day, risperidone 0.8 mg/day, aripiprazole 4 mg/day, and haloperidol 1.6 mg/day. Based on a study by Leucht et al. [9], the high doses for the above APDs were as follows: chlorpromazine 250 mg/day, quetiapine 150 mg/day, olanzapine 7.5 mg/day, risperidone 2 mg/day, aripiprazole 10 mg/day, and haloperidol 4 g/day.

### Statistical analysis

To compare the baseline characteristics between RT and CCRT patients based on the status of APD prescription, we used the Chi-square test for categorical variables and the t-test for continuous variables. Categorical variables were expressed as frequencies (percentages) and the proportion of APD-prescribed patients within each category. Continuous variables were expressed as mean (standard deviation). Among the patients prescribed APD, we examined the proportions of those prescribed during the total period and those prescribed during RT or CCRT, including the proportions of single and multiple prescriptions, as well as the types of APD medications used. We also investigated the timing of APD prescriptions and the proportion of APD prescription doses (low dose/high dose) in monotherapy patients. For patients prescribed APD during RT or CCRT, we analyzed the proportion of APD prescription durations based on 3 months. Additionally, since the study includes patients with various types of cancer, we confirmed the APD prescription rates according to the cancer site during RT treatment.

To assess the mortality risk based on APD prescription status among patients during RT or CCRT, we used the Cox proportional hazards model and estimated the unadjusted and adjusted hazard ratios (HR) and 95% confidence intervals (95% CI). The proportional hazards assumption was checked using a log-minus-log survival plot and time-dependent covariates in the Cox regression model, and it was not violated. The adjusted confounders included age, sex, CCI, medical history, and psychiatric history. Stratified analyses were performed for psychiatric history (total and delirium), prescription time, and prescription duration. A two-sided P-value of < 0.05 was considered statistically significant. All statistical analyses were conducted using SAS version 9.4 (SAS Institute, Cary, NC, USA) and R software version 4.1.3 (R Foundation for Statistical Computing, Vienna, Austria.

## Results

### Frequency of APD use and baseline characteristics

**Fig. 1:**
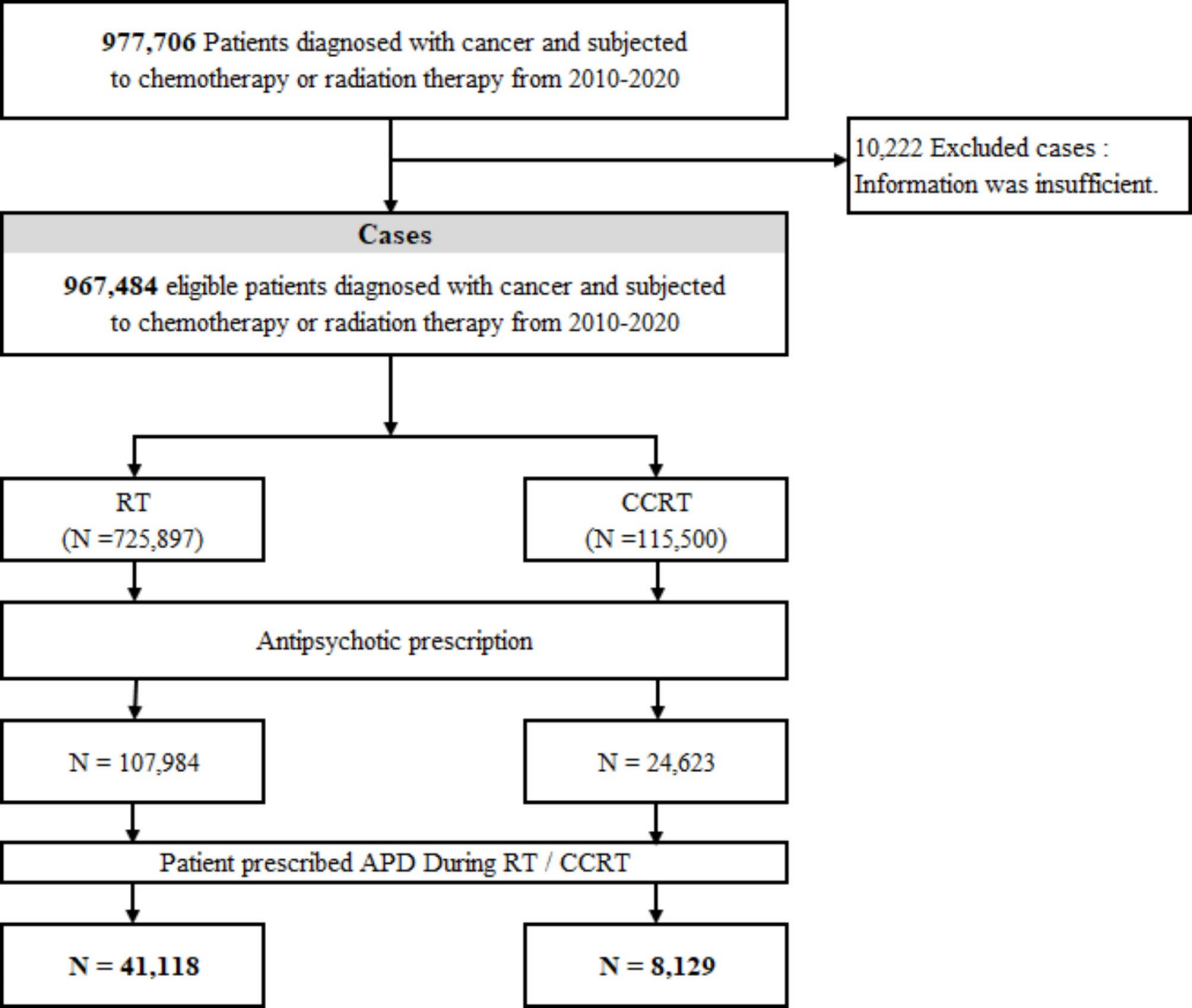
Flow chart of patient selection. RT: radiotherapy, CCRT: concomitant chemo-radiotherapy, APD: antipsychotic drugs

From the NHIS data, a total of 977,706 cancer patients who underwent RT or CTx were identified, and of them, 967,484 patients with sufficient information were analyzed. A total of 725,897 patients received RT, and 115,500 patients received CCRT. Among the patients who received RT, 107,984 (14.9%) took APDs during the entire period, and 41,118 (5.6%) took APDs during RT. Among the patients who received CCRT, 24,623 (21.3%) took APDs during the entire period, and 8,129 (7%) patients took APDs during CCRT. Figure 1 shows the flow chart of patient selection.

Table 1 presents the baseline characteristics and corresponding proportion of APD use in each variable. There was a statistically significant difference in all variables depending on whether APD was used (*p* < 0.0001).

**Table 1:** Baseline characteristics and proportion of antipsychotics use

	RT (*N* = 725,897)			CCRT (*N* = 115,500)			
Category	APD (-) *N* = 684,779	APD (+) *N* = 41,118	Proportion of APD%	APD (-) *N* = 107,371	APD (+) *N* = 8,129	Proportion of APD%	
**Sex**							
Male	277,881 (40.58%)	27,789 (67.58%)	9.09	64,336 (59.92%)	6398 (78.71%)	9.05	
Female	406,898 (59.42%)	13,329 (32.42%)	3.17	43,035 (40.08%)	1731 (21.29%)	3.87	
**Age (Mean**,** SD)**	57.08 (14.19)	64.63 (12.72)		60.28 (11.77)	63.33 (11.03)		
< 30	19,395 (2.83%)	465 (1.13%)	2.34	1002 (0.93%)	50 (0.62%)	4.75	
30–39	53,790 (7.86%)	1047 (2.55%)	1.91	4188 (3.9%)	175 (2.15%)	4.01	
40–49	130,236 (19.02%)	3309 (8.05%)	2.48	13,403 (12.48%)	642 (7.9%)	4.57	
50–59	178,722 (26.1%)	8253 (20.07%)	4.41	29,758 (27.72%)	1895 (23.31%)	5.99	
60–69	157,668 (23.02%)	11,971 (29.11%)	7.06	34,074 (31.73%)	2823 (34.73%)	7.65	
70–79	113,708 (16.61%)	11,834 (28.78%)	9.43	21,585 (20.1%)	2182 (26.84%)	9.18	
≥80	31,260 (4.56%)	4239 (10.31%)	11.94	3361 (3.13%)	362 (4.45%)	9.72	
**CCI (Mean**,** SD)**	3.46 (2.77%)	3.82 (2.88)		3.64 (2.89)	4.17 (3.07)		
0	53,628 (7.83%)	2636 (6.41%)	4.69	8328 (7.76%)	441 (5.43%)	5.03	
1	126,187 (18.43%)	6386 (15.53%)	4.82	19,104 (17.79%)	1210 (14.88%)	5.96	
2	149,736 (21.87%)	7992 (19.44%)	5.07	22,487 (20.94%)	1489 (18.32%)	6.21	
≥ 3	355,228 (51.87%)	24,104 (58.62%)	6.35	57,452 (53.51%)	4989 (61.37%)	7.99	
**Medical history**							
Hypertension (I10-I15)	291,622 (42.58%)	24,386 (59.3%)	7.72	50,101 (46.66%)	4662 (57.35%)	8.51	
Dyslipidemia (E78)	400,651 (58.50%)	28,515 (69.35%)	6.64	62,321 (58.04%)	5566 (68.47%)	8.20	
Diabetes Mellitus (E10-E14)	257,763 (37.64%)	22,935 (55.78%)	8.17	45,920 (42.76%)	4566 (56.17%)	9.04	
Chronic renal Disease (N17-N19)	94,529 (13.8%)	10,884 (26.47%)	10.33	15,400 (14.34%)	1964 (24.16%)	11.31	
Stroke (I60-I69)	31,721 (4.63%)	3862 (9.39%)	10.85	4568 (4.25%)	557 (6.85%)	10.87	
Peripheral Vascular Disease (I70-I79)	155,785 (22.74%)	13,682 (33.27%)	8.07	26,338 (24.52%)	2674 (32.89%)	9.22	
Myocardial Infarction (I21, I22)	18,227 (2.66%)	2321 (5.64%)	11.30	3321 (3.09%)	452 (5.56%)	11.98	
Anemia (D50-53, D55-59)	145,551 (21.25%)	11,904 (28.95%)	7.56	22,131 (20.61%)	2025(24.91%)	8.38	
**Psychiatric History**	181,908 (26.56%)	28,664 (69.71%)	13.61	30,399 (28.31%)	5439 (66.90%)	15.17	
Bipolar disorder (F30, F31)	3797 (0.55%)	5952 (14.4%)	61.05	758 (0.71%)	1073 (13.20%)	58.60	
Psychotic disorder (F20-F29 )	2920 (0.42%)	4073 (9.91%)	58.24	755 (0.70%)	955 (11.74%)	55.84	
Depressive disorder (F32-F34)	126,753 (18.51%)	14,953 (36.36%)	10.55	18,628 (17.35%)	2714 (33.38%)	12.72	
Delirium (F05)	3716 (0.54%)	1749 (4.25%)	32.00	638 (0.59%)	249 (3.06%)	28.07	
Other psychiatric disorders (F00-F99)	98,487(14.38%)	10,834 (26.35%)	9.91	14,591(14.52%)	2214 (27.23%)	14.01	

RT: radiotherapy, CCRT: concomitant chemo-radiotherapy, APD: antipsychotic drugs, CCI: Charlson Comorbidity Index

* Proportion of APD: APD (+) / (APD (-) + APD (+)) *100

** There was a statistically significant difference in all variables depending on whether ADP was used

### Prescription of antipsychotic drugs

For single prescriptions, the most frequently prescribed APDs during RT were quetiapine (64.93%), chlorpromazine (14.24%), olanzapine (11%), risperidone (9.8%), aripiprazole (0.02%), and haloperidol (0.01%). For single prescriptions, the most frequently prescribed APDs during CCRT were quetiapine (44.87%), chlorpromazine (31.62%), olanzapine (16.92%), and risperidone (6.59%). The proportion of patients who received complex prescriptions for APDs during RT was 14%, while the proportion of these patients during CCRT was 9%. The prescribed APDs during RT and CCRT are presented in Table 2.

**Table 2:** Prescribed antipsychotic drugs

	RT		CCRT	
Antipsychotic prescription	Total period *N* = 107,984	during RT *N* = 41,118	Total period *N* = 24,623	during CCRT *N* = 8,129
**Antipsychotic prescription (Duplicate allowed)**	132,946	44,286	30,289	8531
Haloperidol	25 (0.02%)	3 (0.01%)	7 (0.02%)	-
Chlorpromazine	14,765 (11.11%)	5929 (13.39%)	5030 (16.61%)	2556 (29.96%)
Aripiprazole	72 (0.05%)	10 (0.02%)	8 (0.03%)	-
Olanzapine	18,381 (13.83%)	5354 (12.09%)	4506 (14.88%)	1462 (17.14%)
Quetiapine	80,121 (60.27%)	27,406 (61.88%)	16,708 (55.16%)	3798 (44.52%)
Risperidone	19,582 (14.73%)	5584 (12.61%)	4030 (13.31%)	715 (8.38%)
**Simple prescription**	86,808	38,108	19,806	7742
Haloperidol	4 (0.005%)	2 (0.01%)	1 (0.01%)	-
Chlorpromazine	9702 (11.18%)	5427 (14.24%)	3507 (17.71%)	2448 (31.62%)
Aripiprazole	4 (0.005%)	9 (0.02%)	1 (0.01%)	-
Olanzapine	9497 (10.94%)	4191 (11%)	2521 (12.73%)	1310 (16.92%)
Quetiapine	60,658 (69.88%)	24,744 (64.93%)	12,358 (62.4%)	3474 (44.87%)
Risperidone	6943 (8%)	3735 (9.8%)	1418 (7.16%)	510 (6.59%)
**complex**	21,176	3010	4817	387
2	17,803 (84.07%)	2854 (94.82%)	4060 (84.28)	372 (96.12%)
≥ 3	3373 (15.93%)	156 (5.18%)	757 (15.72)	15 (3.88%)

RT: radiotherapy, CCRT: concomitant chemo-radiotherapy

Of the patients who used APDs during RT, while 31% were prescribed APD before RT, 69% were prescribed APD during RT. Only 40% of these patients continued with the APD prescription 60 days after RT. 11% of patients were prescribed APD for more than 3 months and 89% of patients were prescribed APD for less than 3 months. Of the patients who used APDs during RT, 95% were prescribed low-dose APD, and only 5% were prescribed high-dose APD. Of the patients who used APDs during CCRT, 25% received the prescription before RT, and 4% were prescribed high-dose APD. The APD prescription details based on RT and APD doses is presented in Table 3.

**Table 3:** APD prescription details

	RT		CCRT	
	Total patients prescribed APD	Patients prescribed APD during RT	Total patients prescribed APD	Patients prescribed APD during CCRT
**APD prescription time**	*N* = 107,984	*N* = 41,118	*N* = 24,623	*N* = 8,129
**Pre-RT**	28,085 (26.01%)	12,655 (30.78%)	5,262 (21.37%)	2,061 (25.35%)
**During RT**	28,463 (26.36%)	28,463 (69.22%)	6,068 (24.64%)	6,068 (74.65%)
**After RT**	51,436 (47.63%)	16,329 (39.71%)	13,293 (35.06%)	2,990 (36.78%)
60 ~ 365days	21,725 (20.12%)	10,979 (26.70%)	6,146 (16.21%)	2,201 (27.07%)
> 365days	29,711 (27.51%)	5,350 (13.01%)	7,147 (18.85%)	789 (9.70%)
**APD duration**	-		-	
Within 3 months		36,511 (88.8%)		7,542 (92.78%)
Over 3months		4,607 (11.2%)		587 (7.22%)
APD dose *	*N* = 86,808	*N* = 38,108	*N* = 19,806	*N* = 7,742
Low dose	79,168 (91.2%)	36,140 (94.8%)	17,984(90.8%)	7,299 (94.3%)
High dose	7,830 (11.8%)	1,968 (5.2%)	1,822 (9.2%)	443 (5.7%)

RT: radiotherapy, CCRT: concomitant chemo-radiotherapy, APD: antipsychotic drugs, * Based on monotherapy

### *APD use* according to the cancer site

Supplementary Table 1 presents the frequency and proportion of APD use during RT based on cancer sites. Duplicates were allowed for patients with more than one diagnostic code. The five cancer sites with the highest number of patients on APDs during RT were the lungs (21.64%), breast (5.09%), colorectum (4.44%), liver (4.17%), and head and neck (1.01%). On the other hand, the five cancer sites with the highest proportion of APD use during RT were the lungs (14.87%), brain (14.27%), esophagus (12.95%), ureter and bladder (4.17%), and stomach (11.02%).

### Influence of antipsychotics on the survival of cancer patients

**Fig. 2:**
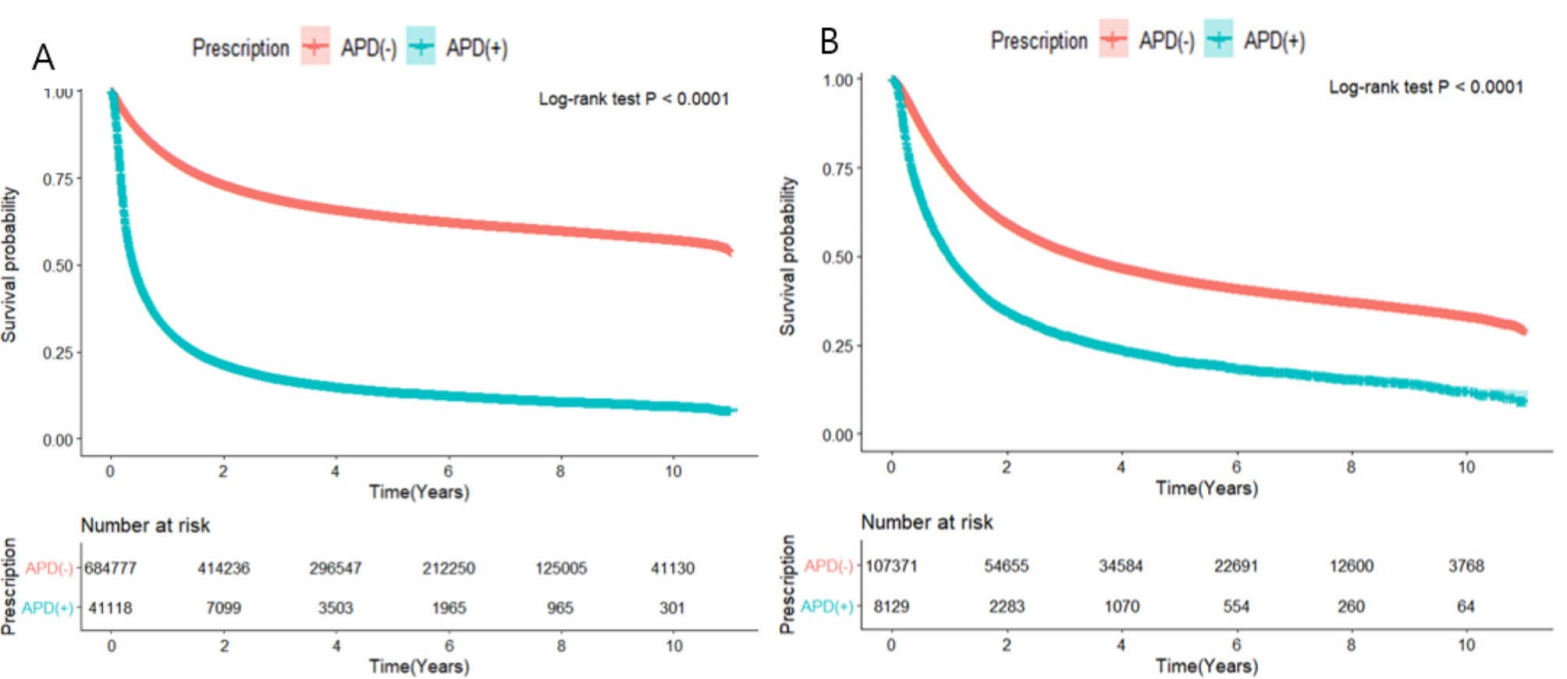
Survival analysis of cancer patients according to antipsychotic drugs during radiotherapy or concomitant chemo-radiotherapy. RT: radiotherapy, CCRT: concomitant chemo-radiotherapy

Table 4 presents the survival analyses based on the use of APD during RT or CCRT and APD prescription details. Using the Cox proportional hazard regression analysis, we estimated the risk of mortality based on APD use during RT without adjustment and with adjustment by sex, age, CCI, and medical history. Both in the unadjusted and adjusted analyses, using APD during RT was significantly associated with higher mortality (unadjusted HR: 5.15, 95% CI: 5.09–5.21; adjusted HR: 3.45, 95% CI: 3.4–3.49, p-value < 0.0001). Both in the time-fixed and time-dependent Cox proportional hazard regression analysis, using APD during RT was significantly associated with higher mortality and these results are presented in supplementary Table 2. Figure 2A presents the survival analyses based on the use of APD during RT.

**Table 4:** Cox proportional hazard regression analysis on the risk of mortality in patients received radiotherapy

Category	Incidence rate				Unadjusted		Adjusted	
	N	Case	Person years	Incidence rate	**HR (95% CI)**	**p-value**	**HR (95% CI)**	**p-value**
**During RT**								
**Non APD**	684,779	234,081	2781532.8	84.16	ref (1.00)		ref (1.00)	
**APD**	41,118	33,854	49991.56	677.19	5.25 (5.19–5.31)	< 0.0001	3.58 (3.54–3.63)	< 0.0001
**Psychiatric (-)** ^**a**^	12,454	11,050	12,855.87	859.53	ref (1.00)		ref (1.00)	
**Psychiatric (+)** ^**a**^	28,664	22,804	37,135.70	614.07	0.77(0.75–0.79)	< 0.0001	0.88(0.84–0.93)	< 0.0001
Delirium^b^	1,749	1,451	1,943.18	746.71	ref (1.00)		ref (1.00)	
Excluding delirium^b^	26,945	21.353	38,048.38	606.75	0.93(0.88–0.98)	0.0093	0.90(0.85–0.96)	0.0005
**Prescription Time** ^**a**^								
Pre RT	12,655	7,725	20,526.42	376.34	ref (1.00)		ref (1.00)	
During RT	28,463	20,224	23,147.42	873.70	1.84(1.79–1.89)	< 0.0001	1.32(1.27–1.37)	< 0.0001
Non after RT	24,789	27,949	49991.56	639.95	ref (1.00)		ref (1.00)	
After RT	16,329	12,905	8,317.72	934.67	1.02(0.99–1.05)	0.3066	1.05(1.02–1.08)	0.0016
60 ~ 365days	10,0.979	8,786	6,894.84	773.47	ref (1.00)		ref (1.00)	
> 365days	5,350	4,119	2,422.88	1,489.23	0.82(0.80–0.86)	< 0.0001	0.72(0.68–0.74)	< 0.0001
**Prescription duration** ^**a**^								
< 3 month	37,649	31,451	43,806.67	717.95	ref (1.00)		ref (1.00)	
≥ 3 month	3,469	2,403	6,184.89	388.53	0.63 (0.6–0.65)	< 0.0001	0.80 (0.76–0.83)	< 0.0001
**Prescription dose** ^**a**^								
Low dose	36,140	29,905	42912.61	696.88	ref (1.00)		ref (1.00)	
High dose	1,968	1,293	4302.05	300.55	0.6 (0.57–0.63)	< 0.0001	0.87 (0.82–0.92)	< 0.0001
**During CCRT**								
**Non APD**	107,371	58,040	351870.47	164.95	ref (1.00)		ref (1.00)	
**APD**	7,742	5,671	13828.8	410.09	2.1 (2.04–2.16)	< 0.0001	1.72 (1.67–1.77)	< 0.0001
**Psychiatric (-)** ^**c**^	2,303	31,815	219,860.5	144.71	ref (1.00)		ref (1.00)	
**Psychiatric (+)** ^**c**^	5,439	32,207	146,440.4	219.93	0.99 (0.94–1.05)	0.7420	0.93 (0.81–1.07)	0.3072
Delirium ^d^	249	193	381.69	505.65	ref (1.00)		ref (1.00)	
Excluding delirium ^d^	7,880	5,789	14,048.79	412.06	0.97(0.89–1.02)	0.0862	0.96(0.88–1.01)	0.0772
**Prescription Time** ^**c**^								
Pre RT ^**c**^	2061	1421	3705.66	383.47	ref (1.00)		ref (1.00)	
During RT ^**c**^	6068	4064	9481.18	428.64	1.13(1.06–1.20)	0.0001	0.97(0.90–1.06)	0.5520
Non after RT ^**c**^	5451	4064	9481.18	428.64	ref (1.00)		ref (1.00)	
After RT ^**c**^	2990	497	1243.63	399.64	0.96(0.88–1.06)	0.4099	0.94(0.86–1.03)	0.2103
**Prescription duration** ^**c**^								
< 3 month ^**c**^	7542	5580	13286.87	419.96	ref (1.00)		ref (1.00)	
≥ 3 month ^**c**^	587	402	1143.61	351.52	0.84 (0.76–0.93)	0.0005	0.90 (0.80-1.00)	0.045
**Prescription dose** ^**c**^								
Low dose ^**c**^	7,299	5,384	12887.47	417.77	ref (1.00)		ref (1.00)	
High dose ^**c**^	443	287	941.34	304.88	0.78 (0.69–0.87)	< 0.0001	0.9 (0.8–1.02)	0.1026

HR: Hazard ratios, RT: radiotherapy, CCRT: concomitant chemo-radiotherapy, APD: antipsychotic drugs, Adjusted HR: Survival analysis adjusted for Sex, Age, CCI and medical history and psychiatric history, a: in the patients who used APDs during RT, b: in patients who used APDs during RT with psychiatric history, c: in the patients who used APDs during CCRT, d: in patients who used APDs during CCRT with psychiatric history

Of the patients who used APDs during RT, the patients with psychiatric history had lower mortality compared to those without psychiatric history (adjusted HR: 0.88, 95% CI: 0.84–0.93, p-value < 0 0.0001). Among the patients who used APDs during RT with psychiatric history, the patients without delirium had lower mortality compared to those with delirium (adjusted HR: 0.90, 95% CI: 0.85–0.96, p-value 0.0005). Of the patients who used APDs during RT, analysis based on prescription time showed higher mortality in patients started APD during RT compared to those started APD pre-RT (adjusted HR: 1.32, 95% CI: 1.27–1.37, p-value < 0 0.0001). Analysis based on APD prescription duration showed lower mortality in patients were prescribed APD for more than 3 months compared to those were prescribed APD for less than 3 months (adjusted HR: 0.80, 95% CI: 0.76–0.83, p-value < 0.0001). Analysis based on APD dose showed lower mortality in patients on high-dose APD compared to those on low-dose APD (adjusted HR: 0.87, 95% CI: 0.82–0.92, p-value < 0.0001).

Using APD during CCRT was significantly associated with a higher mortality rate (adjusted HR: 1,72, 95% CI: 1.67–1.77, p-value < 0 0.0001). Analysis based on APD prescription duration showed lower mortality in patients were prescribed APD for more than 3 months compared to those were prescribed APD for less than 3 months (adjusted HR: 0.90, 95% CI: 0.80-1.00, p-value = 0.045). Analysis based on APD prescription time and APD dose showed comparable mortality. Figure 2B present the survival analyses based on the use of APD during CCRT.

Among the APDs used during RT, chlorpromazine and quetiapine were associated with the lowest and highest mortality rates, respectively. Analyses based on APD dose found that high-dose chlorpromazine (HR: 0.65, 95% CI: 0.5–0.85, p-value = 0.002) and risperidone (HR: 0.85, 95% CI: 0.74–0.93, p-value = 0.001) were associated with lower mortality, whereas low-dose olanzapine (HR: 1.22, 95% CI: 1.12–1.33, p-value < 0.001) was associated with lower mortality. The mortality rates in patients receiving high or low doses of quetiapine were comparable. The risk of mortality based on APD doses according to prescribed APDs dose is presented in supplementary Table 2.

## Discussion

This study aimed to evaluate APD use in adult cancer patients who underwent RT and assess its effect on survival. To the best of our knowledge, this study is the first to report the prevalence of APD use in cancer patients in South Korea and the first to report the prevalence of APD use among adult cancer patients receiving RT in Korea.

Patients diagnosed with cancer often have concurrent psychiatric disorders in addition to general psychological distress [10]. A National Register study compared the combined incidence of mental disorders in cancer patients and individuals without cancer and found a higher incidence in the former group [11]. While approximately one-third of patients with cancer experience comorbid psychiatric disorders, it is estimated that they are recognized and treated in less than half of these patients [12]. Psychotropic medications are used to treat mental health disorders and are of five main types: antidepressants, anti-anxiety medications, stimulants, mood stabilizers, and APDs. APDs can reduce or relieve symptoms of psychosis, such as delusions (false beliefs) and hallucinations (seeing or hearing something that is not there). APDs, also known as neuroleptics, are primarily used to treat various psychiatric conditions, including schizophrenia, bipolar disorder, psychotic depression, and Alzheimer’s disease. In addition, APDs are also used to reduce anxiety in anxiety disorders and tics in Tourette syndrome. APDs are classified as either first-generation or typical antipsychotics, which act as dopamine receptor antagonists, or as second-generation or atypical antipsychotics, which act as serotonin-dopamine antagonists [13]. In this study, we analyzed the most commonly prescribed APDs in clinical practice, including typical APDs, such as haloperidol and chlorpromazine, and atypical APDs, such as aripiprazole, quetiapine, olanzapine, and risperidone.

The most common psychiatric disorders accompanying cancer patients include adaptation, anxiety, depression, physical symptoms, substance use, and post-traumatic stress disorders [14]. Cancer patients with psychosis are not common, with an incidence rate less than 5% [15]. However, psychotic symptoms (e.g., delusions or hallucinations) may develop in cancer patients with no pre-existing psychotic disorder, secondary to CTx agents such as chlorambucil, hydroxyurea, ifosfamide, asparaginase, corticosteroids, interferon-alpha, interleukin-2, and procarbazine [16, 17]. This study found that the proportion (5.6%) of Korean patients using APDs during RT was higher than that of Chinese (1.4%), Brazilian (1.67%), and Dutch (4.7%) cancer patients [15, 18, 19]. The difference in proportions can be attributed to the fact that the other studies analyzed all cancer patients, whereas our study focused on cancer patients who underwent RT.

The proportion of patients using APDs during RT was more than twice in men (9.09%) than in women (3.17%). This may be because psychotic disorders are more common in men [20]. The proportion of patients using APDs during RT increased with age; 11.94% of those over 80 years used APDs during RT. This may be due to the increase in the incidence of delirium in old age, because quetiapine, which has been shown to be the most used APD in our study, is used to treat delirium [21]. CCI was higher in patients using APDs during RT or CCRT, and the proportion of patients using APDs increased as CCI increased. Although there were significant differences, the proportions of patients with hypertension, diabetes mellitus, chronic renal disease, stroke, and anemia were comparable among the APD and non-APD users. In contrast, the proportions of patients with psychiatric history were higher in APD patients than in non-APD patients.

The lung was the most frequent cancer site in patients who used APD during RT. Moreover, patients with lung cancer had a high proportion of APD users. Patients with breast cancer accounted for 5.09% of patients who used APD during RT, but the proportion of APD users with breast cancer was only 1.97%. Despite the relatively low number of patients with breast cancer on APDs, this cancer type is the most commonly treated with RT in Korea. Therefore, the breast was analyzed as a relatively common cancer site for patients on APDs during RT [22]. On the other hand, smaller proportions of patients with cancer of the brain (3.3%), esophagus (2.94%), stomach (2.03%), and the ureter and bladder (1.32%) used APDs during RT. However, the proportions of APD patients with cancer in these sites were high (14.27, 12.95, 11.02, and 12.48%, respectively). A meta-analysis by Jared et al. reported that individuals with psychotic disorders had a higher incidence of breast, esophageal, colorectal, testicular, uterine, and cervical cancer [23]. They also had 22% higher odds of metastases at diagnosis compared to those without psychotic disorders [23]. Although it is not possible to directly compare the patients analyzed in their study with those in our study, the high rate of APD use during RT in cancer patients with metastases may be associated with the higher incidence of metastasis observed in patients with psychotic disorders.

Among adult cancer patients, the most commonly prescribed APD during RT was quetiapine (61.88%). Quetiapine is a commonly prescribed atypical APD for schizophrenia, depression, and bipolar disorder. It is frequently used for delirium and behavioral and psychological symptoms in dementia. Recently, Bhat et al. observed that quetiapine increases the radio-sensitivity of glioblastoma (GBM) cells in GBM mice models. They showed that combining quetiapine with irradiation led to the increased expression of genes related to cholesterol synthesis in resistant glioma cells, and the addition of atorvastatin, a 3-hydroxy-3-methyl-glutaryl-coenzyme A reductase inhibitor, enhanced the efficacy of quetiapine [7]. Furthermore, several recent studies have reported the presumptive mechanism of action behind the anti-cancer effects of APDs [24]. However, a meta-analysis revealed that individuals diagnosed with psychotic disorders had a pooled age-adjusted risk ratio of 1.08 (95% CI: 1.01–1.15) for all cancers compared with those without psychotic disorders [23]. Individuals with psychotic disorders also had 22% higher (95% CI: 2–46%) odds of metastases at diagnosis compared to those without psychotic disorders. Hence, the presence of concurrent psychiatric disorders in cancer patients may be associated with increased mortality. Among patients with esophageal cancer who underwent surgery and had no previous history of psychiatric disorders, mortality was found to be 70% higher in patients with psychiatric disorders than in those without psychiatric disorders [25]. In our study, patients receiving APD during RT had a worse survival rate than those who did not, which does not necessarily mean that APD does not have anticancer or radiosensitizer effects. The patients included in our study could have had different reasons for undergoing RT, reasons for taking APD, and treatment durations. In addition, patient responses following RT could not be confirmed from our data; therefore, the radiosensitivity of the APD could not be confirmed. Future studies should investigate these items and further test this hypothesis moving forward.

In our study, the diagnosis of psychiatric history was classified and analyzed to the reason for the use of APD in patients who used APD during RT. However, unfortunately, not all patients who used APD were accompanied by a psychiatric diagnostic code. In our study, 30% of patients were prescribed APD without psychiatric diagnosis, possibly due to the culture of reluctance to diagnose psychiatric patients in South Korea. In fact, a report stated that 7% percent of those affected by mental illness sought psychiatric help and 12.1% of people with diagnosed illnesses seeking treatment in Korea [26]. The cause of this situation may be Koreans have a relatively high level of internalized stigma for mental illness and discrimination based on mental health conditions.

Our study found a higher mortality rate (HR 3.45, 95% CI: 3.4–3.49) in patients using APDs during RT and a higher risk of mortality compared to previous studies [23, 25]. This is probably because we included only cancer patients who used APDs during RT and not cancer patients diagnosed with psychiatric disorders. Of the patients who used APDs during RT, the patients with psychiatric history had lower mortality compared to those without psychiatric history. Among the patients who used APDs during RT with psychiatric history, the patients without delirium had lower mortality compared to those with delirium. It is likely that patients who used APDs during RT without psychiatric history have been accompanied by psychiatric disorders such as delirium. The proportion of haloperidol or aripiprazole use during RT was very low. Moreover, the proportion of patients taking high-dose APD was as low as 5%. In the analysis based on APD dose, higher mortality rates were seen in patients on low-dose APD than those on high-dose APD. This suggests the possibility that the APD was used during RT to mitigate symptoms of delirium. APDs are used for the pharmacological management of delirium and should be administered at the lowest appropriate dose [27]. The high mortality in patients who started APD during RT and patients who used APD for less than 3 months is also presumed to be related to delirium. It is presumed that the reason that APD had to be started during RT and that APD had to be used for less than 3 months is due to the clinical course of delirium. It is possible that the reason for having to start APD during RT and having to use APD for less than 3 months was due to the clinical course of delirium. Although the proportion of cancer patients in our study with the diagnosis code of delirium was very low, it is estimated that patients with delirium are much higher in reality. In our study, about 30% patients who took APD during RT had no specified psychiatric diagnosis, and many of whom were presumed to have been accompanied by delirium. This is because delirium in cancer patients is often designated with other psychiatric diagnostic codes or the code is not specified in clinical practice. Delirium is the most common neurological disease in cancer patients and is more commonly observed in older patients. Studies show that 22‒44% of patients with cancer experience delirium and that the incidence rises to 87% in the final days of life [28]. In our study, patients who took quetiapine, the most commonly used APD during RT, had the highest mortality rates with no differences between the low- and high-dose groups. Quetiapine, which has a very low incidence of extrapyramidal side effects, is often the primary choice for delirious elderly patients. Although we cannot be completely sure if most patients who took APD during RT in our study had delirium, this could be one of the reasons for the high mortality rate. In admitted terminal cancer patients, higher mortality rates have been reported in patients with delirium than without it [29]. Further studies on the effects of APDs on cancer treatment and prognosis are needed.

There are some limitations to our study. First, because our database contained all cancer patients with significant heterogeneity by cancer site and the NHIS database lacks information on disease conditions such as tumor stage, which has a significant effect on prognosis, the effect of APD use during RT on survival rates could not be analyzed. Second, our database included only 5-FU and CDDP regimens, the two most commonly used CTx regimens used in CCRT. Therefore, our analysis might not fully represent the use of APD in all patients undergoing CCRT. For accurate analysis of APD use during CCRT, further investigation using a dataset that includes all CTx drugs is required. Finally, no clear reason for using APD was identified in all patients included in this study. This study was based on medical claims, not medical chart reviews. Given the limited clinical presentations in the NHIS dataset, non-claims information or other factors affecting APD use were excluded from the analyses.This can be a variable that confuses outcomes by not sufficiently reflecting the underline status of patients who had to use APD. Therefore, we should be careful when interpreting the results.

## Conclusions

Only 5.6% of patients who received RT and 7% of those who received CCRT took APDs during the treatment. Quetiapine was the most frequently prescribed APD during RT. The use of APD during RT may adversely affect survival. However, further studies are required to determine the effects of APDs on cancer patients.

## Electronic supplementary material

Below is the link to the electronic supplementary material.

Supplementary Material 1

## Data Availability

This study was based on the Health Insurance Review and Assessment Service (HIRA) database, which encompasses medical expense claim data for the entire South Korean population. The authors do not own these data and hence are not permitted to share them in the original form.

## Abbreviations

APD: Antipsychotic drug
RT: Radiotherapy
CTx: Chemotherapy
CCRT: Concomitant chemo-radiotherapy
NHIS: National Health Insurance Service
5-FU: 5-fluorouracil (5-FU)
CDDP: Cisplatin
CCI: Charlson Comorbidity Index
HR: Hazard ratio
CI: Confidence interval
CINV: Chemotherapy induced nausea and vomiting
