# Supplementary material for: Use of antipsychotic drugs during radiotherapy in adult cancer patients in Korea: a nationwide retrospective cohort study based on the national health insurance service database
Source: Radiat Oncol. 2024 Nov 29;19:171. doi: 10.1186/s13014-024-02558-8 (PMC11607900; doi:10.1186/s13014-024-02558-8)
Supplement: Supplementary file 1 — Supplementary Material 1 [file 13014_2024_2558_MOESM1_ESM.docx]

**Supplementary table 1. Frequency and proportion of antipsychotics use during radiotherapy according to cancer site**

|  | **Total** | | | **Male** | | | **Female** | | |
| --- | --- | --- | --- | --- | --- | --- | --- | --- | --- |
| **Category** | **APD (-)** | **APD (+)** | **Proportion of APD%** | **APD (-)** | **APD (+)** | **Proportion of APD%** | **APD (-)** | **APD (+)** | **Proportion of APD%** |
| **Cancer site** |  |  |  |  |  |  |  |  |  |
| Brain (C70-C71) | 13105 (1.58) | 2182 (3.3) | 14.27 | 7293 (2.06) | 1392 (3.07) | 16.03 | 5812 (1.23) | 790 (3.8) | 11.97 |
| Breast (C50) | 166865 (20.13) | 3361 (5.09) | 1.97 | 327 (0.09) | 24 (0.05) | 6.84 | 166538 (35.12) | 3337 (16.07) | 1.96 |
| Colorectum (C18-C20) | 47391 (5.72) | 2934 (4.44) | 5.83 | 30886 (8.71) | 1992 (4.4) | 6.06 | 16505 (3.48) | 942 (4.54) | 5.40 |
| Esophagus (C15-C17) | 13033 (1.57) | 1939 (2.94) | 12.95 | 11842 (3.34) | 1823 (4.02) | 13.34 | 1191 (0.25) | 116 (0.56) | 8.88 |
| Gynecologic (C51-58) | 32675 (3.94) | 1228 (1.86) | 3.62 | - | - |  | 32675 (6.89) | 1228 (5.91) | 3.62 |
| Head and neck  (C00-C14, C30-C32) | 33662 (4.06) | 2652 (4.01) | 7.30 | 26787 (7.56) | 2207 (4.87) | 7.61 | 6875 (1.45) | 445 (2.14) | 6.08 |
| Liver (C22) | 35098 (4.24) | 2755 (4.17) | 7.28 | 27971 (7.89) | 2282 (5.04) | 7.54 | 7127 (1.5) | 473 (2.28) | 6.22 |
| Lung (C34) | 81846 (9.88) | 14297 (21.64) | 14.87 | 60806 (17.15) | 11624 (25.66) | 16.05 | 21040 (4.44) | 2673 (12.87) | 11.27 |
| Metastasis (C77-C79) | 104504 (12.61) | 17769 (26.9) | 14.53 | 53299 (15.03) | 12375 (27.32) | 18.84 | 51205 (10.8) | 5394 (25.98) | 9.53 |
| Non-Hodgkin’s lymphoma (C82-C88) | 14414 (1.74) | 1340 (2.03) | 8.51 | 8060 (2.27) | 850 (1.88) | 9.54 | 6354 (1.34) | 490 (2.36) | 7.16 |
| Pancreas (C25) | 10613 (1.28) | 1054 (1.6) | 9.03 | 6066 (1.71) | 703 (1.55) | 10.39 | 4547 (0.96) | 351 (1.69) | 7.17 |
| Prostate (C61) | 33798 (4.08) | 1645 (2.49) | 4.64 | 33798 (9.53) | 1645 (3.63) | 4.64 | - | - |  |
| Stomach (C16) | 10817 (1.31) | 1339 (2.03) | 11.02 | 7542 (2.13) | 1029 (2.27) | 12.01 | 3275 (0.69) | 310 (1.49) | 8.65 |
| Thyroid (C73) | 153324 (18.5) | 1111 (1.68) | 0.72 | 33843 (9.55) | 319 (0.7) | 0.93 | 119481 (25.2) | 792 (3.81) | 0.66 |
| Ureter and bladder  (C66-C67) | 6094 (0.74) | 869 (1.32) | 12.48 | 4720 (1.33) | 718 (1.59) | 13.20 | 1374 (0.29) | 151 (0.73) | 9.90 |
| Others | 71502 (8.63) | 9585 (14.51) | 11.82 | 41284 (11.64) | 6313 (13.94) | 13.26 | 30218 (6.37) | 3272 (15.76) | 9.77 |
| Total | 828,741 | 66,060 | 7.38 | 354,524 | 45,296 | 11.33 | 474,217 | 20,764 | 4.19 |

APD: antipsychotic drugs

* Proportion of APD: APD (+) / (APD (-) + APD (+)) *100

** There was a statistically significant difference in all variables depending on whether ADP was used.

**Supplementary table 2. Risk of mortality according to APD prescription using Time-fixed Cox model and Time-dependent Cox model**

| Category | Case/N | Incidence rate | Time-**fixed** Cox Model | | Time-**dependent** Cox Model | |
| --- | --- | --- | --- | --- | --- | --- |
|  |  | (1000 PY) | HR (95% CI) | p-value | HR | p-value |
| Patients prescribed APD during RT | | | | | | |
| Non APD | 21,392/39,378 | 160.01 | ref (1.00) |  | ref (1.00) |  |
| APD | 16,532/19,689 | 649.81 | 3.41 (3.33-3.49) | <.0001 | 3.41 (3.33-3.49) | <.0001 |
| Patients prescribed APD during CCRT | | | | | | |
| Non APD | 5,426/9,224 | 186.49 | ref (1.00) |  | ref (1.00) |  |
| APD | 3,503/4,612 | 421.67 | 1.99 (1.90-2.09) | <.0001 | 2.01 (1.91-2.1) | <.0001 |

RT: radiotherapy, CCRT: concomitant chemo-radiotherapy, APD: antipsychotic drugs

**Supplementary table 3. Cox proportional hazards regression analysis on the risk of mortality according to prescribed APD dose in patients who used APDs during RT.**

| **category** | **Incidence rate** | | | | **Unadjusted** | | **Adjusted** | |
| --- | --- | --- | --- | --- | --- | --- | --- | --- |
|  | N | Case | Person years | Incidence rate | **HR (95% CI)** | **p-value** | **HR (95% CI)** | **p-value** |
| **Prescription dose ^a^** | | | | |  |  |  |  |
| Low dose | 36,140 | 29,905 | 42912.61 | 696.88 | ref (1.00) |  | ref (1.00) |  |
| High dose | 1,968 | 1,293 | 4302.05 | 300.55 | 0.6 (0.57-0.63) | <.0001 | 0.87 (0.82-0.92) | <.0001 |
| **Chlorpromazine ^a^** | | |  |  |  |  |  |  |
| Low dose | 5350 | 4386 | 9330.69 | 470.06 | ref (1.00) |  | ref (1.00) |  |
| High dose | 77 | 55 | 229.56 | 239.59 | 0.65 (0.5-0.85) | 0.002 | 0.65 (0.5-0.85) | 0.002 |
| **Olanzapine ^a^** | | |  |  |  |  |  |  |
| Low dose | 3265 | 2321 | 4570.2 | 507.86 | ref (1.00) |  | ref (1.00) |  |
| High dose | 926 | 691 | 1416.48 | 487.83 | 1.1 (1.01-1.2) | 0.023 | 1.22 (1.12-1.33) | <.0001 |
| **Quetiapine ^a^** | | |  |  |  |  |  |  |
| Low dose | 24682 | 20777 | 25423.07 | 817.25 | ref (1.00) |  | ref (1.00) |  |
| High dose | 62 | 53 | 73.62 | 719.91 | 1.14 (0.87-1.49) | 0.357 | 1.1 (0.84-1.45) | 0.474 |
| **Risperidone ^a^** | | |  |  |  |  |  |  |
| Low dose | 2843 | 2421 | 3588.65 | 674.63 | ref (1.00) |  | ref (1.00) |  |
| High dose | 892 | 490 | 2571.66 | 190.54 | 0.41 (0.37-0.45) | <.0001 | 0.83 (0.74-0.93) | 0.001 |

HR: Hazard ratios , RT: radiotherapy, CCRT: concomitant chemo-radiotherapy, APD: antipsychotic drugs, Adjusted HR: Survival analysis adjusted for Sex, Age, CCI and medical history and psychiatric history
